# Participation in college laboratory research apprenticeships among students considering careers in medicine

**DOI:** 10.3402/meo.v20.27231

**Published:** 2015-06-23

**Authors:** Dorothy A. Andriole, Donna B. Jeffe, Robert H. Tai

**Affiliations:** 1 Department of Surgery, Washington University School of Medicine, St Louis, MO, USA; 2 Department of Medicine, Washington University School fo Medicine, St Louis, MO, USA; 3 Department of Curriculum, Instruction and Special Education, Curry School of Education, University of Virginia, Charlottesville, VA, USA

**Keywords:** student research apprenticeships, medical-school admissions, medical-student diversity, pre-medical education, medical-student selection criteria

## Abstract

**Objective:**

We sought to determine the prevalence of college laboratory research apprenticeship (CLRA) participation among students considering medical careers and to examine the relationship between CLRA participation and medical-school acceptance among students who applied to medical school.

**Methods:**

We used multivariate logistic regression to identify predictors of: 1) CLRA participation in a national cohort of 2001–2006 Pre-Medical College Admission Test (MCAT) Questionnaire (PMQ) respondents and 2) among those PMQ respondents who subsequently applied to medical school, medical-school acceptance by June 2013, reporting adjusted odds ratios (aOR) and 95% confidence intervals (95% CI).

**Results:**

Of 213,497 PMQ respondents in the study sample (81.2% of all 262,813 PMQ respondents in 2001–2006), 72,797 (34.1%) reported CLRA participation. Each of under-represented minorities in medicine (URM) race/ethnicity (vs. white, aOR: 1.04; 95% CI: 1.01–1.06), Asian/Pacific Islander race/ethnicity (vs. white, aOR: 1.20; 95% CI: 1.17–1.22), and high school summer laboratory research apprenticeship (HSLRA) participation (aOR: 3.95; 95% CI: 3.84–4.07) predicted a greater likelihood of CLRA participation. Of the 213,497 PMQ respondents in the study sample, 144,473 (67.7%) had applied to medical school and 87,368 (60.5% of 144,473 medical-school applicants) had been accepted to medical school. Each of female gender (vs. male, aOR: 1.19; 95% CI: 1.16–1.22), URM race/ethnicity (vs. white, aOR: 3.91; 95% CI: 3.75–4.08), HSLRA participation (aOR: 1.11; 95% CI: 1.03–1.19), CLRA participation (aOR: 1.12; 95% CI: 1.09–1.15), college summer academic enrichment program participation (aOR: 1.26; 95% CI: 1.21–1.31), and higher MCAT score (per point increase, aOR: 1.31; 95% CI: 1.30–1.31) predicted a greater likelihood of medical-school acceptance.

**Conclusions:**

About one-third of all PMQ respondents had participated in CLRAs prior to taking the MCAT, and such participation was one of the several variables identified that were independently associated with medical-school acceptance.

The National Science Foundation (NSF) and the National Institutes of Health (NIH), among other federal and private institutions including many colleges and medical schools, fund programs at the college level to provide students with opportunities to participate in research (1–4). There are some college research programs explicitly intended to promote the participation of students from under-represented groups in research-related careers (5–7); many other college research programs are designed to provide all interested students with opportunities to gain research experience.

Participants in college laboratory research programs who graduate from college may subsequently pursue a variety of advanced educational opportunities, including enrollment in PhD degree or master's degree programs in the biomedical sciences and enrollment in medical school or in other allied health professions schools. Many students who consider careers in medicine participate in laboratory research opportunities during college, and medical schools may consider an applicant's research experiences in the admissions process (8). However, the prevalence of participation in college laboratory research opportunities among students considering careers in medicine and the relationship between such participation and medical-school applicants’ acceptance to medical school have not been described.

We conducted a national, retrospective cohort study of individuals considering careers in medicine to explore the prevalence of participation in college laboratory research opportunities among this population, to determine whether such participation was associated with applying to medical school and to test the hypothesis that such participation would be positively associated with medical-school acceptance.

## Method

For this study, we operationally defined students considering careers in medicine as Medical College Admission Test (MCAT) examinees (9). To identify MCAT examinees who had participated in college laboratory research opportunities during college, we utilized data from the Association of American Medical Colleges (AAMC) Pre-MCAT Questionnaire (PMQ), a lengthy questionnaire that includes a variety of demographic, experiential, and attitudinal items (10). PMQ completion is voluntary. The PMQ was administered to MCAT examinees before the initial MCAT attempt through 2013 and has since been administered to examinees after the initial MCAT attempt, as the Post-MCAT Questionnaire (10, 11). With Institutional Review Board approval, we constructed a database of individualized, de-identified records for all individuals who had completed the PMQ in 2001–2006. The content of the PMQ changes annually to some extent as some new items are added and other items are revised or deleted. We excluded PMQ respondents prior to 2001 because of differences in items of interest on the PMQ prior to 2001. We excluded PMQ respondents after 2006 because, in more recent years, the PMQ was offered to only a portion of MCAT registrants (12). The database included records of individual-level data from the AAMC Data Warehouse, the repository for all data collected by the AAMC pertaining to individual MCAT examinees, medical-school applicants, acceptees, matriculants, enrollees, and graduates. The individual-level data provided by the AAMC included responses to selected items on the PMQ, initial-attempt MCAT scores, and each of medical-school application, acceptance, and matriculation, with all records updated through June 9, 2013. A lengthy period of follow-up was necessary, as the numbers of medical-school applicants and matriculants continue to accrue among MCAT examinees for many years after the initial MCAT date.

PMQ variables in our study included gender and race/ethnicity (categorized as under-represented minorities in medicine [URM, including Black, Hispanic and Native American/Alaska Native], Asian/Pacific Islander [PI], other/multiple races/unknown, and white). We also included selected responses to a PMQ item about participation in various types of programs intended to prepare high school or college students for professional schooling or careers in medicine or related fields. Respondents could select more than one program; we included responses for each of the following programs: summer academic enrichment program for college students, high school summer laboratory research apprenticeship (HSLRA), and college laboratory research apprenticeship (CLRA). Based on responses to the two research apprenticeship choices (HSLRA and CLRA), we created a four-category variable for participation in laboratory research apprenticeship programs (HSLRA+CLRA, HSLRA only, CLRA only, and none). We also included responses to a PMQ item about participation in ‘Paid or volunteer work in hospitals, medical clinics, or labs’ (hereafter referred to as, ‘Health professions exposure indicator’) and an item about participation in ‘Honors clubs not specific to my undergraduate major [such as Phi Kappa Alpha, National Honor Society]’ (hereafter referred to as, ‘Honors society indicator’).

We used responses to two PMQ items about the highest education level obtained by each parent to create a seven-category variable for parents’ highest education level: 1) data not provided for both parents, 2) at least one parent is<high school (HS) graduate and neither parent is a HS graduate, 3) at least one parent is a HS graduate but not college graduate or higher, 4) at least one parent is a college graduate but neither parent is an advanced-degree holder, 5) at least one parent is a master's degree holder but neither parent holds a higher degree, 6) at least one parent has a doctoral degree but neither parent is a physician, and 7) at least one parent is a physician (MD or DO-degree holder), which was the reference group. We included parent education, because parents’ education may have an effect on their children's academic achievement (13, 14) and because parents also may positively influence their children's decisions about pursuing careers in medicine; about 70% of students entering US medical schools reported that parents have a positive/very positive influence on their decision to study medicine rather than pursue another career (15).

The Carnegie Classifications of undergraduate institutions (16) attended by PMQ respondents were provided by the AAMC. Using these data, we created a six-category variable for Carnegie Classification of PMQ respondents’ undergraduate institutions including: 1) research universities with very high research activity (the reference group), 2) research universities with high research activity and doctoral/research universities (other research universities), 3) masters colleges/universities, 4) Baccalaureate Arts and Sciences (A&S) colleges, 5) all other Carnegie Classifications of undergraduate institutions, and 6) Carnegie Classification not specified.

The AAMC provided data for each PMQ respondent's initial MCAT scores. We computed a composite score as the sum of the Verbal Reasoning, Biological Sciences, and Physical Sciences sub-scores. The AAMC also provided indicators for each PMQ respondent's status regarding whether or not the individual had applied to at least one U.S. Liaison Committee for Medical Education (LCME)-accredited medical school by June 2013, had been accepted to at least one US LCME-accredited medical school by June 2013, and had matriculated at a US LCME-accredited medical school by June 2013. Based on these records, we created dichotomous variables for medical-school application (yes vs. no), acceptance (yes vs. no), and matriculation.

### Statistical analysis

We report descriptive statistics for each variable examined in association with CLRA participation. We also report results of multivariate logistic regression models to identify independent predictors of: 1) CLRA participation (including all PMQ respondents in the study sample), 2) medical-school application (including all PMQ respondents), and 3) medical-school acceptance (including only those respondents in the study sample who applied to medical school). We report adjusted odds ratios (aOR) and 95% confidence intervals (CI) from each of these three regression models. We ran each of the medical-school application and the medical-school acceptance models first with only MCAT score to estimate the shared variance between MCAT score and each outcome of interest, reporting the Nagelkerke R^2^ for each model, and then we added the remaining predictors of interest as a block in addition to MCAT scores, examining the increase in Nagelkerke R^2^ in each of the two models. All tests were performed using SPSS version 20.0.0.1 (IBM SPSS, Inc., Chicago, IL, USA).

## Results

Our database included individualized records for all 262,813 individuals who had initially completed at least part of the PMQ in 2001–2006. Of these 262,813 PMQ respondents, we excluded from our study 12,381 (4.7%) respondents who did not subsequently complete the MCAT and 36,935 (14.1%) respondents who did not complete all PMQ items of interest. Thus, our final study sample included 213,497 PMQ respondents (81.2% of all 2001–2006 PMQ respondents).

Of these 213,497 PMQ respondents, 72,797 (34.1%) had participated in CLRAs. Descriptive statistics for our study sample, grouped by CLRA participation, are shown in Table 1.

**Table 1:** Multivariate logistic regression model identifying variables associated with College Laboratory Research Apprenticeship (CLRA) participation among 2001–2006 Pre-Medical College Admission Test (MCAT) questionnaire respondents (N=213,497)

Variables	Total N=213,497 (%)^a^	CLRA participation, N=72,797 (%)^b^	No CLRA participation, N=140,700 (%)^b^	aOR (95% CI)^c^
Gender				
Men	97,974 (45.9)	33,308 (34.0)	64,666 (66.0)	1.00 (Reference)
Women	115,523 (54.1)	39,489 (34.2)	76,034 (65.8)	1.01 (0.99–1.02)
Race/ethnicity				
White	126,616 (59.3)	40,894 (32.3)	85,722 (67.7)	1.00 (Reference)
Under-represented in medicine	34,539 (16.2)	11,842 (34.3)	22,697 (65.7)	**1.04 (1.01–1.06)**
Asian/Pacific Islander	42,811 (20.1)	16,861 (39.4)	25,950 (60.6)	**1.20 (1.17–1.22)**
Other/multiple/unknown	9,531 (4.5)	3,200 (33.6)	6,331 (66.4)	1.02 (0.97–1.07)
Highest level of parents’ education				
Neither parent graduated from high school	5,385 (2.5)	1,729 (32.1)	3,656 (67.9)	**0.92 (0.87–0.99)**
At least one parent graduated from high school	47,571 (22.3)	14,378 (30.2)	33,193 (69.8)	**0.94 (0.91–0.97)**
At least one parent graduated from college	60,219 (28.2)	19,739 (32.8)	40,480 (67.2)	1.03 (1.00–1.06)
At least one parent has master's degree	45,165 (21.2)	16,499 (36.5)	28,666 (63.5)	**1.16 (1.12–1.20)**
At least one parent has (non-medical) doctoral degree	25,561 (12.0)	10,222 (40.0)	15,339 (60.0)	**1.26 (1.22–1.31)**
At least one parent is a physician	29,596 (13.9)	10,230 (34.6)	19,366 (65.4)	1.00 (Reference)
Carnegie classification of undergraduate institution				
Research universities with very high research activity	90,830 (42.5)	34,690 (38.2)	56,140 (61.8)	1.00 (Reference)
Not specified	32,130 (15.0)	8,896 (27.7)	23,234 (72.3)	**0.64 (0.62–0.66)**
Other institutions	6,666 (3.1)	1,823 (27.3)	4,843 (72.7)	**0.68 (0.64–0.72)**
Baccalaureate A&S colleges	19,595 (9.2)	7,607 (38.8)	11,988 (61.2)	**1.08 (1.05–1.12)**
Master's colleges/ universities	30,630 (14.3)	9,276 (30.3)	21,354 (69.7)	**0.78 (0.76–0.81)**
Other research universities	33,646 (15.8)	10,505 (31.2)	23,141 (68.8)	**0.80 (0.77–0.82)**
High school summer laboratory research apprenticeship				
No	191,921 (89.9)	58,835 (30.7)	133,086 (69.3)	1.00 (Reference)
Yes	21,576 (10.1)	13,962 (64.7)	7,614 (35.3)	**3.95 (3.84–4.07)**

aOR, adjusted odds ratio; CI, confidence interval; A&S, arts and sciences.

a Percentage of column total.

b Percentage of row total for each variable characteristic.

c aORs with 95% CIs that do not include 1.00 are statistically significant and are given in bold.

Table 1 also shows the results of the multivariate logistic regression model to identify variables independently associated with CLRA participation. Compared to the reference groups shown in the table for each variable, respondents who were URM or Asian/PI race/ethnicity, participated in a HSLRA, attended Baccalaureate A&S institutions, and whose parents’ highest level of education was masters’ degree or doctoral (non-medical) degree were more likely to report CLRA participation. Respondents who attended institutions with unspecified Carnegie Classifications or classified as other, master's colleges/universities, or other research universities, and whose parents’ highest level of education was HS graduation or less than HS graduation were less likely to report CLRA participation.

Of the 213,497 PMQ respondents in our study sample, 144,473 (67.7%) had applied to at least one US LCME-accredited medical school.

Table 2 shows the characteristics of our study sample, grouped by medical-school application status and the results of the multivariate logistic regression model to identify independent predictors of medical-school application among PMQ respondents in our study. In addition to all variables shown in Table 2, we controlled for PMQ year in the multivariate regression model; PMQ year was not independently associated with medical-school application (aOR=1.00, 95% CI=1.00–1.01). As shown in Table 2, compared to the reference groups shown in the table for each variable, respondents who were URM race/ethnicity, Asian/PI, and race/ethnicity reported CLRA participation, honors society participation, health professions exposure, and college summer enrichment program participation, and had higher MCAT scores were more likely to have applied to medical school. Respondents who were women, attended undergraduate institutions with unspecified Carnegie Classification or classified as other and master's colleges/universities, and who did not have at least one parent who was a physician were less likely to have applied to medical school. In the logistic regression model including MCAT alone, Nagelkerke R^2^=18.9; in the multivariate model with all variables of interest included, Nagelkerke R^2^=39.7.

**Table 2:** Multivariate logistic regression model identifying variables associated with medical-school application by June 2013 among 2001–2006 Pre-Medical College Admission Test (MCAT) respondents (N=213,497)

	Total N=213,497	Applied to medical school, N=144,473	Did not apply to medical school, N=69,024	
				
Variable	Mean (SD)	Mean (SD)	Mean (SD)	aOR (95% CI)^a^
MCAT score^b^	24.9 (6.7)	26.6 (6.1)	21.3 (6.5)	**1.18 (1.18–1.18)**
	N (%)^c^	N (%)^d^	N (%)^d^	
Gender				
Men	97,974 (45.9)	69,585 (71.0)	28,389 (29.0)	1.00 (Reference)
Women	115,523 (54.1)	74,888 (64.8)	40,635 (35.2)	**0.95 (0.93–0.97**)
Race/ethnicity				
White	126,616 (59.3)	87,451 (69.1)	39,165 (30.9)	1.00 (Reference)
Under-represented in medicine	34,539 (16.2)	22,935 (66.4)	11,604 (33.6)	**2.34 (2.26–2.42)**
Asian/Pacific Islander	42,811 (20.1)	28,290 (66.1)	14,521 (33.9)	**1.16 (1.12–1.19)**
Other/multiple/unknown	9,531 (4.5)	5,797 (60.8)	3,734 (39.2)	0.96 (0.91–1.01)
Highest level of parents’ education				
Neither parent graduated from high school	5,385 (2.5)	3,044 (56.5)	2,341 (43.5)	**0.72 (0.67–0.77)**
At least one parent graduated from high school	47,571 (22.3)	27,706 (58.2)	19,865 (41.8)	**0.61 (0.59–0.64)**
At least one parent graduated from college	60,219 (28.2)	38,776 (64.4)	21,443 (35.6)	**0.67 (0.65–0.70)**
At least one parent has master's degree	45,165 (21.2)	32,474 (71.9)	12,691 (28.1)	**0.75 (0.72–0.78)**
At least one parent has (non-medical) doctoral degree	25,561 (12.0)	19,739 (77.2)	5,822 (22.8)	**0.85 (0.81–0.89)**
At least one parent is a physician	29,596 (13.9)	22,734 (76.8)	6,862 (23.2)	1.00 (Reference)
Carnegie classification				
Research universities with very high research activity	90,830 (42.5)	73,291 (80.7)	17,539 (19.3)	1.00 (Reference)
Not specified	32,130 (15.0)	7,948 (24.7)	24,182 (75.3)	**0.08 (0.08–0.08)**
Other institutions	6,666 (3.1)	3,982 (59.7)	2,684 (40.3)	**0.84 (0.80–0.89)**
Baccalaureate A&S colleges	19,595 (9.2)	15,386 (78.5)	4,209 (21.5)	1.02 (0.97–1.06)
Master's colleges/universities	30,630 (14.3)	20,003 (65.3)	10,627 (34.7)	**0.88 (0.85–0.91)**
Other research universities	33,646 (15.8)	23,863 (70.9)	9,783 (29.1)	1.00 (0.96–1.03)
Laboratory research apprenticeships				
None	133,086 (62.3)	85,407 (64.2)	47,679 (35.8)	1.00 (Reference)
HSLRA	7,614 (3.6)	5,024 (66.0)	2,590 (34.0)	0.97 (0.92–1.03)
CLRA	58,835 (27.6)	44,006 (74.8)	14,829 (25.2)	**1.10 (1.07–1.13)**
HSLRA+CLRA	13,962 (6.5)	10,036 (71.9)	3,926 (28.1)	1.02 (0.97–1.07)
College summer academic enrichment program				
No	187,521 (87.8)	125,708 (67.0)	61,813 (33.0)	1.00 (Reference)
Yes	25,976 (12.2)	18,765 (72.2)	7,211 (27.8)	**1.27 (1.23–1.32)**
Honors society indicator				
No	137,670 (64.5)	85,261 (61.9)	52,409 (38.1)	1.00 (Reference)
Yes	75,827 (35.5)	59,212 (78.1)	16,615 (21.9)	**1.64 (1.60–1.68)**
Health professions exposure indicator				
No	60,721 (28.4)	37,071 (61.1)	23,650 (38.9)	1.00 (Reference)
Yes	152,776 (71.6)	107,402 (70.3)	45,374 (29.7)	**1.33 (1.30–1.36)**

aOR, adjusted odds ratio; CI, confidence interval; A&S, arts and sciences; HSLRA, high school summer laboratory research apprenticeship; CLRA, college laboratory research apprenticeship; SD, standard deviation.

a aORs with 95% CIs that do not include 1.00 are statistically significant and are given in bold.

b OR>1.000 indicates greater likelihood for each unit increase in composite MCAT score.

c Percentage of column total.

d Percentage of row total for each characteristic.

Table 3 shows the characteristics of the 144,473 medical-school applicants, grouped by medical-school admission; 87,368 (60.5%) of these applicants were ultimately accepted to at least one US LCME-accredited medical school. Table 3 also shows the results of the multivariate logistic regression model to identify independent predictors of medical-school acceptance. In addition to all variables shown in Table 3, we controlled for PMQ year in the model; more recent PMQ year was independently associated with a lower likelihood of medical-school acceptance (aOR=0.89, 95% CI=0.89–0.90). Compared to the reference groups shown in the table for each variable, medical-school applicants who were women and were URM race/ethnicity reported each of HSLRA, CLRA, or HSLRA+CLRA participation, Honors society participation, health professions exposure, and college summer enrichment program participation, attended Baccalaureate A&S colleges, and had higher MCAT scores were more likely to have been accepted to medical school. Applicants who were Asian/PI or other/multiple races/unknown race/ethnicity attended undergraduate institutions with unspecified Carnegie Classification or classified as master's colleges/universities, and who did not have at least one parent who was a physician were less likely to have been accepted to medical school. In the logistic regression model including MCAT alone, Nagelkerke R^2^=33.2; in the multivariate model with all variables of interest included, Nagelkerke R^2^=41.3. Of the 87,368 applicants accepted to medical school, 85,711 (98.1%) were US LCME-accredited medical-school matriculants.

**Table 3:** Multivariate logistic regression model identifying variables associated with medical-school acceptance by June 2013 among 2001–2006 Pre-Medical College Admission Test (MCAT) respondents who applied to medical school (N=144,473)

	Total N=144,473	Accepted, N=87,368	Not accepted, N=57,105	
				
Variable	Mean (SD)	Mean (SD)	Mean (SD)	aOR (95% CI)^a^
MCAT score^b^	26.6 (6.1)	29.1 (5.0)	22.9 (5.6)	**1.31 (1.30–1.31)**
	N (%)^c^	N (%)^d^	N (%)^d^	
Gender				
Men	69,585 (48.2)	43,862 (63.0)	25,723 (37.0)	1.00 (Reference)
Women	74,888 (51.8)	43,506 (58.1)	31,382 (41.9)	**1.19 (1.16–1.22)**
Race/ethnicity				
White	87,451 (60.5)	55,141 (63.1)	32,310 (36.9)	1.00 (Reference)
Under-represented in medicine	22,935 (15.9)	13,333 (58.1)	9,602 (41.9)	**3.91 (3.75–4.08)**
Asian/Pacific Islander	28,290 (19.6)	16,241 (57.4)	12,049 (42.6)	**0.81 (0.79–0.84)**
Other/multiple/unknown	5,797 (4.0)	2,653 (45.8)	3,144 (54.2)	**0.55 (0.52–0.59)**
Highest level of parents’ education				
Neither parent graduated from high school	3,044 (2.1)	1,399 (46.0)	1,645 (54.0)	**0.76 (0.69–0.83)**
At least one parent graduated from high school	27,706 (19.2)	13,508 (48.8)	14,198 (51.2)	**0.60 (0.58–0.63)**
At least one parent graduated from college	38,776 (26.8)	21,981 (56.7)	16,795 (43.3)	**0.65 (0.63–0.68)**
At least one parent has a master's degree	32,474 (22.5)	20,719 (63.8)	11,755 (36.2)	**0.74 (0.70–0.77)**
At least one parent has a (non-medical) doctoral degree	19,739 (13.7)	13,996 (70.9)	5,743 (29.1)	**0.86 (0.82–0.91)**
At least one parent is physician	22,734 (15.7)	15,765 (69.3)	6,969 (30.7)	1.00 (Reference)
Carnegie classification				
Research universities with very high research activity	73,291 (50.7)	49,339 (67.3)	23,952 (32.7)	1.00 (Reference)
Not specified	7,948 (5.5)	3,376 (42.5)	4,572 (57.5)	**0.36 (0.34–0.38)**
Other institutions	3,982 (2.8)	1,807 (45.4)	2,175 (54.6)	0.99 (0.91–1.07)
Baccalaureate A&S colleges	15,386 (10.6)	10,390 (67.5)	4,996 (32.5)	**1.22 (1.17–1.28)**
Master's colleges/universities	20,003 (13.8)	9,413 (47.1)	10,590 (52.9)	**0.86 (0.82–0.89)**
Other research universities	23,863 (16.5)	13,043 (54.7)	10,820 (45.3)	1.03 (1.00–1.07)
Laboratory research apprenticeships				
None	85,407 (59.1)	48,331 (56.6)	37,076 (43.4)	1.00 (Reference)
HSLRA	5,024 (3.5)	3,036 (60.4)	1,988 (39.6)	**1.11 (1.03–1.19)**
CLRA	44,006 (30.5)	29,234 (66.4)	14,772 (33.6)	**1.12 (1.09–1.15)**
HSLRA+CLRA	10,036 (6.9)	6,767 (67.4)	3,269 (32.6)	**1.21 (1.14–1.28)**
College summer academic enrichment program				
No	125,708 (87.0)	75,773 (60.3)	49,935 (39.7)	1.00 (Reference)
Yes	18,765 (13.0)	11,595 (61.8)	7,170 (38.2)	**1.26 (1.21–1.31)**
Honors society indicator				
No	85,261 (59.0)	47,751 (56.0)	37,510 (44.0)	1.00 (Reference)
Yes	59,212 (41.0)	39,617 (66.9)	19,595 (33.1)	**1.81 (1.76–1.86)**
Health professions exposure indicator				
No	37,071 (25.7)	21,296 (57.4)	15,775 (42.6)	1.00 (Reference)
Yes	107,402 (74.3)	66,072 (61.5)	41,330 (38.5)	**1.07 (1.04–1.10)**

aOR, adjusted odds ratio; CI, confidence interval; A&S, arts and sciences; HSLRA, high school laboratory research apprenticeship; CLRA, college laboratory research apprenticeship; SD, standard deviation.

a aORs with 95% CIs that do not include 1.00 are statistically significant and are given in bold.

b OR>1.000 indicates greater likelihood for each unit increase in composite MCAT score.

c Percentage of column total.

d Percentage of row total for each characteristic (i.e., number of accepted applicants [or number of not accepted applicants]/row total number) for each variable.

## Discussion

About one-third of PMQ respondents in our study sample reported CLRA participation, which was associated with a greater likelihood of applying to medical school and, among applicants, with a greater likelihood of being accepted to medical school, as hypothesized. We discuss our observations about CLRA participation in the context of the current educational environment for students aspiring to careers in medicine.

### 

#### Variables associated with CLRA participation

Among all PMQ respondents in our study sample, URM and Asian/PI race/ethnicity predicted a greater likelihood of CLRA participation. Thus, although lack of racial/ethnic diversity in the biomedical research workforce is an issue of national concern (17), non-white race/ethnicity did not appear to be a barrier to CLRA participation in this cohort. A relatively small proportion of PMQ respondents in our study had reported HSLRA participation, but such participation predicted a much greater likelihood of CLRA, providing support for the role of opportunities for the exposure to research early in the educational continuum as among the means to promote student involvement in biomedical research at the post-secondary school level.

Our observations regarding associations between parent education and CLRA participation might reflect to some extent a relationship between family socioeconomic status and CLRA participation (18). Opportunities for college students to participate in laboratory research can include both paid and unpaid positions. Children of parents with relatively lower family incomes (generally parents with less than college-level education) may have to work during college and thus may be less likely than children of more highly educated (and affluent) parents (generally parents with higher levels of education), to be able to participate in unpaid, or relatively low-paying, extracurricular activities such as a CLRA. The relationship between parent education and CLRA participation also might reflect a generally greater awareness among parents who had at least completed college, regardless of income, about how to access potential opportunities for their children to participate in research programs. Respondents whose parents held master's degrees or other, non-medical doctoral degrees were more likely than respondents with physician parents to report CLRA participation, suggesting that the type of education parents received could influence their children's access to and/or choice of extracurricular activities to pursue during college and after, as we report below.

The observed associations between Carnegie Classification of undergraduate institutions and CLRA participation suggest that opportunities to participate in CLRAs may vary substantially among different types of institutions. As respondents who attended Baccalaureate A&S colleges were more likely than respondents who had attended very high research activity universities to have reported CLRA participation, we speculate that Baccalaureate A&S colleges might have particularly robust, accessible undergraduate research programs; alternatively, students at these colleges might benefit from particularly strong advising, mentoring, and support to seek out such opportunities if they are interested in exploring science-related career opportunities and/or careers in medicine.

#### Variables associated with medical-school application

A majority of PMQ respondents in our study sample ultimately applied to medical school. MCAT score alone accounted for most of the total shared variance in medical-school application that we observed. Respondents who reported participating in CLRA only were more likely to apply to medical school compared with respondents who participated in neither HSLRA nor CLRA (aOR 1.10). However, participation in both HSLRA and CLRA was not associated with a greater likelihood of medical-school application. Students who had reported multiple research apprenticeships starting in high school might comprise a group with particularly strong research interests and might have pursued other more research-focused career paths (e.g., PhD degrees) rather than medicine. The association between Carnegie Classification and medical-school application suggests that there might be differences in access to, and quality of, pre-medical advising programs across different types of undergraduate institutions. That URM students were particularly likely to apply to medical school may reflect extensive, ongoing efforts at the medical institutions nationally to encourage URM students to pursue careers in medicine (19). We also observed that PMQ respondents who did not have at least one physician parent were less likely to apply to medical school. It was previously reported that children of parents with advanced degrees are substantially over-represented, and children of parents without college degrees are substantially under-represented, among medical-school applicants (20); our findings indicated that, among children of parents with advanced degrees, there are also differences in representation among medical-school applicants in association with type of advanced degrees held by parents.

#### Variables associated with medical-school acceptance

As we had hypothesized, there was a positive, independent association between CLRA participation and medical-school acceptance. Thus, CLRA participation appears to provide a benefit to students aspiring to careers in medicine in getting accepted to medical school, independent of their MCAT scores. However, the magnitude of the observed relationship was modest (aOR 1.12, indicating approximately a 12% higher likelihood of acceptance) and somewhat less than the magnitude of the relationship that we observed between acceptance to medical school and a unit increase in MCAT score.

As medical schools place considerable importance on academic performance criteria in admissions, it was not surprising that MCAT score alone accounted for so much of the total shared variance that we observed in medical-school acceptance and that the ‘honors society indicator’ also was positively and independently associated with medical-school acceptance. Our findings regarding the associations between medical-school acceptance and Carnegie Classification of undergraduate institutions are consistent with the observations from a previous study (21), which reported that admission rates were highest among applicants from Baccalaureate Arts & Sciences Colleges and Research universities with very high research activity. These authors speculated that differences may exist across different types of institutions in the ability to access important resources, such as knowledge of the application process and pre-medical educational programs (21). Our findings regarding the association between medical-school acceptance and parent education also extends findings from other reports that, compared to their representation among medical-school applications, a nationally representative sample of high school sophomores whose parents had college, masters, or other advanced degrees were relatively over-represented among medical-school matriculants, whereas children of parents who had not completed college were relatively under-represented (20). Physician parents, in particular, may be especially helpful in guiding their children through the medical-school application process and accessing useful resources.

We examined medical-school acceptance in association with gender and race/ethnicity in a model that also included MCAT scores and other predictor variables of interest. The greater likelihood of medical-school acceptance of women (vs. men) and URM (vs. white) applicants that we observed in our multivariate regression models might reflect the impact of other, unmeasured variables that may be part of a holistic medical-school admissions process (including evaluation of an applicant's characteristics observed during the interview process, letters of recommendation, and other variables not included in our models) (22).

Our observations about the association of CLRA participation with medical-school acceptance also contribute to the understanding of potential ‘positive spill-over’ effects of such college laboratory research experiences among students who subsequently seek to pursue careers in medicine (5). Previous work has found that participation in research during college is associated with reduced risk of delays in medical-school graduation due to academic difficulties (23) and with suboptimal outcomes, such as medical-school withdrawal or dismissal (24). In addition, in a national cohort study of medical-school graduates, CLRA participation was positively and independently associated with appointment to a full-time position in academic medicine (25).

### Strengths and limitations

Our study has several strengths. Our database included individualized data for a national cohort of all of the 2001–2006, first-time PMQ respondents. We had a minimum of 6 years of follow-up for all individuals in our cohort, and information about medical-school application and acceptance outcomes were based on primary source rather than self-reported data. Our database included 262,813 PMQ respondents from 2001 to 2006, most of whom completed the MCAT; only 12,381 did not have MCAT scores. Published AAMC data indicate that there were 370,113 MCAT examinees in 2001–2006 (26–31). As about 12% of MCAT examinees repeat the examination in a given year (8), the number of first-time MCAT examinees in 2001–2006 can be estimated at 325,699 (88% of 370,113). Thus, our database of PMQ respondents likely included about 77% (250,432/325,699) of all first-time MCAT examinees in 2001–2006, and our final study sample likely included about 65.6% (213,497/325,699) of all first-time MCAT examinees in 2001–2006.

Our study also has some limitations. As a retrospective cohort study, causality cannot be inferred from any of the associations we have reported. Studies using an experimental design (ideally with randomization) would be needed to test the effect of CLRA on medical-school application and acceptance. Furthermore, data regarding participation in CLRAs, as well as the other types of programs intended to prepare high school or college students for professional schooling or careers in medicine (e.g., HSLRA and summer academic enrichment program for college students), were based on self-reported data on the PMQ. As our database comprised completely de-identified data, we could not verify the accuracy of these specific PMQ responses at an individual-respondent level. Also, we did not have information about the duration, quality, or scope of the CLRA programs (or the HSLRA programs) in which PMQ respondents in our study sample may have participated. There are a variety of federally funded research programs for college students (1, 2, 6) and a wide range of institutionally sponsored research programs; thus, findings for any specific laboratory research program may differ from our observations in this national cohort. Similarly, we did not have information about the particular types of college summer academic enrichment programs in which PMQ respondents in our study sample may have participated. However, our findings that participation in college summer academic enrichment programs was independently associated with a greater likelihood of each of medical-school application and medical-school acceptance are aligned with recently reported findings for the Robert Wood Johnson Summer Medical and Dental Education Program (SMDEP), which is a summer academic enrichment program for diverse college students aspiring to careers in medicine and/or dentistry (32). Compared to non-program participants matched for gender, race/ethnicity, and parent education level (among other characteristics), SMDEP participants from those sites that offered the Medical Education Program component (but not the Dental Education Program component) were about 12% more likely to apply to medical school and about 9% more likely to matriculate in medical school (32). Differences in study design between our cohort study and the SMDEP study may have contributed to the lower magnitude of the relationship between program participation and each of medical-school application and medical-school matriculation that was observed for SMDEP participants: whereas our reference group for college summer academic enrichment program participants comprised PMQ respondents who did not report participation in any such program, the matched comparison group for the SMDEP participants may have included individuals who participated in other types of college summer academic enrichment programs, as the authors of the study had noted (32).

We also note that MCAT scores released to us for analysis were first-attempt scores only; MCAT examinees may retake the examination (8). Similarly, the medical-school acceptance data released to us for analysis were cumulative acceptances over the duration of our study period, not acceptance data for a single application cycle year. Published AAMC data for the applicant pool in 2003–2012 indicate that, of the 406,746 applicants during this 10-year period, there were 303,509 first-time applicants (75.1%); thus, about 25% of applicants were repeat applicants (33). This limitation is a particularly important consideration in the context of the greater likelihood of medical-school acceptance that we observed among URM applicants compared to white applicants. Racial/ethnic differences in students’ persistence in re-applying to medical school if they were not initially accepted have been reported with Black/African American students and Hispanic students more likely than white students to re-apply to medical school if they were not initially accepted (12). Thus, our observations may reflect, at least in part, racial/ethnic differences in likelihood of re-application. Differences in the number and types of medical schools to which PMQ respondents in our study applied could also impact their likelihood of medical-school acceptance; however, this information was not provided to us. Finally, we also did not have information about the characteristics of individual medical schools, their admissions criteria, and how these criteria may be weighed, which vary considerably among medical schools in selecting applicants for admission to their schools. Thus, there were several unmeasured variables which could contribute to our understanding of factors associated with medical-school application and medical-school acceptance.

## Conclusions

In our national cohort study, about one-third of all students considering medical careers had participated in CLRAs prior to taking the MCAT, and CLRA participation was independently associated with medical-school acceptance. Broadly, our findings regarding the academic progress of students aspiring to careers in medicine who participate in research programs during high school and college may be of interest to the organizations and institutions (including many medical schools) involved in funding and administering these programs. Our findings regarding variables associated with medical-school application and acceptance may also be of particular interest to individuals and organizations involved in efforts to provide resources and opportunities to a diverse pool of students aspiring to careers in medicine (20).
